# Single-port thoracoscopic rib resection: a case report

**DOI:** 10.1186/1749-8090-9-49

**Published:** 2014-03-15

**Authors:** Chang-Lun Huang, Ching-Yuan Cheng, Ching-Hsiung Lin, Bing-Yen Wang

**Affiliations:** 1 Division of Thoracic Surgery, Department of Surgery, Changhua Christian Hospital, No. 135, Nanxiao St, Changhua, Taiwan; 2 Institute of Medicine, Chung Shan Medical University, Taichung, Taiwan; 3 School of Medicine, National Yang-Ming University, Taipei, Taiwan

**Keywords:** Uniportal, Single-port, Thoracoscopic, Rib resection

## Abstract

We describe a method of single-port thoracoscopic rib resection using a Gigli saw. Rib resection is typically performed with a large skin incision and soft tissue dissection. Some authors have described a thoracoscopic approach for rib resection from the inner side of the chest wall instead of the outside to decrease pain and improve quality of life. A 41-year-old Chinese male received single-port thoracoscopic rib resection with satisfactory recovery. We present the technique, which may expand the indications of single-port thoracoscopic procedures.

## Background

Growing evidence has suggested that video-assisted thoracic surgery is an alternative to conventional thoracotomy for some patients. Most thoracic surgeons use 3 or 4 incisions to perform thoracoscopic surgery. With advances in endoscopic instrumentation, some thoracoscopic surgical procedures are being performed with only a single incision. The applications of single-port thoracoscopic surgery include wedge resection of the lung and treatment of pleural, mediastinal, and chest wall diseases [1]. However, thoracoscopic rib resection via a single incision has not been reported. Herein, we describe single-port thoracoscopic rib resection using a Gigli saw.

## Case presentation

**Figure 1:**
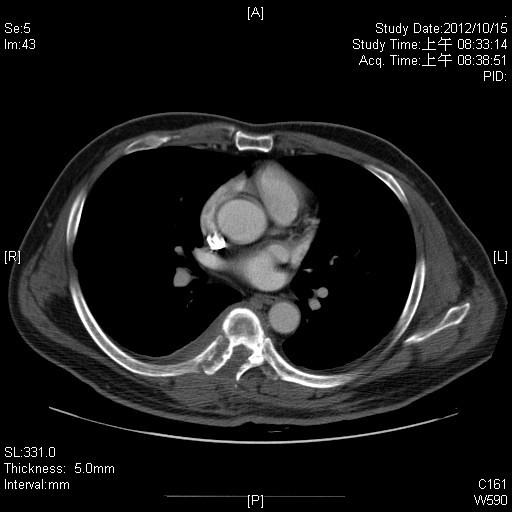
Chest computed tomography scan revealed osteolytic lesions in the right 7th rib with surrounding soft tissue swelling.

A 41-year-old Chinese male presented with lower back pain and bilateral lower leg weakness for 1 month. Chest computed tomography (CT) showed osteolytic lesions over the right 7^th^ rib and adjacent thoracic vertebral body costovertebral junction (Figure 1). Whole body bone scan revealed increased activity at the posterior right 7^th^ rib. CT-guided core biopsy was performed of the paraspinal tissue adjacent to the thoracic spine at the level of the 7^th^ rib, and the pathologic result revealed inflammation. Thus, 7^th^ rib resection was performed for a diagnosis.

### Technique

**Figure 2:**
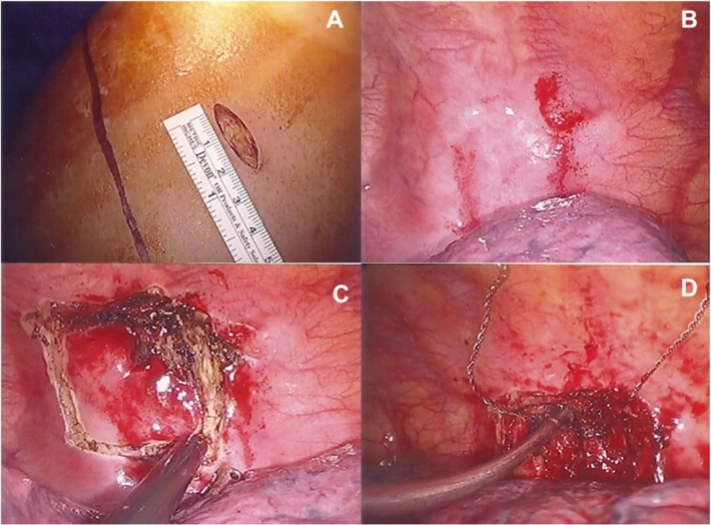
**Intraoperative pictures of single-port thoracoscopic rib resection. (A)** Single-port incision in the 6^th^ intercostal space at the anterior axillary line. **(B)** Thickening pleura under the right 7^th^ rib. **(C)** The parietal pleura under the 7^th^ rib was opened by electrical hook. **(D)** Thoracoscopic view during partial resection of the rib with a Gigli saw.

The patient was placed in the left lateral decubitus position, and general anesthesia and double lumen intubation was performed to achieve unilateral ventilation. The single port was placed in the 6^th^ intercostal space at the anterior axillary line (Figure 2A), and a wound protector was placed. Both the operator and assistant directing the thoracoscope stood on the anterior side of patient. A 10-mm 30° thoracoscope was placed at the posterior side of the incision and other working instruments at the anterior side. Upon examination, thickened pleura under the 7^th^ rib was found (Figure 2B). During most of the procedure, the thoracoscope was fixed at the posterior edge of wound protector to avoid interference with the endoscopic instruments. The parietal pleura under the 7^th^ rib was opened by electrical hook (Figure 2C). Periosteal tissue at proximal and distal end of target area of 7^th^ rib was dissected. The intercostal vessels were coagulated and cut with the electrical hook. Detachment of the soft tissue around the rib was confirmed by using endoscopic forceps. The lower margin of 7^th^ rib was looped with a Gigli saw (Figure 2D). The ends of the Gigli saw were held with 2 clamp forceps, and the 7^th^ rib was transected. The upper margin of 7^th^ rib was transected in the same manner. A 5-cm segment of the rib was resected, and a bag was inserted into thoracic cavity through the port for removal of the rib. One chest tube was inserted via the port wound after removal of the instruments. The operative time was 110 minutes and blood loss was minimal. The final pathologic report reveaed acute and chronic inflammation. The tissue culture grew Saphylococcus aures and adequte antibiotics was administered. The chest drain was removed on the second post-operative day without complication.

## Discussion

Despite the expanding indications for video-assisted thoracic surgery, there have been few reports of chest wall resection and isolated rib resection, and in some cases specific instruments are needed [2]. Recently, Nakagiri et al. [3] described thoracoscopic rib resection using a Gigli saw, which is widely used for the resection of other bones. Rocco et al. [4] also a Gigli saw for rib resection, and further performed reconstruction with a titanium plate to prevent lung herniation. In their reports, however, multiple ports were used. Although reports of thoracoscopic surgery performed with single incision are increasing [5], single-port thoracoscopic rib resection has not been reported.

As described in Rocco’s studies and reviews [1,5], single-port thoracoscopic surgical resections can decrease postoperative pain and hospital stay when compared with conventional 3-port procedures. We perform single-port thoracoscopic rib resection successfully. In our patient, the target area for rib resection was located at the posterior paraspinal region. The port was designed for using the Gigli saw in a perpendicular direction, which allowed the target rib to be most effectively resected. If the target rib is located in a lateral or anterior aspect, grasping instruments could be used to change the direction of the Gigli saw. Thoracoscopes with smaller diameter (5 mm or 3 mm) may offer more space for instruments manipulation or decreased the size of wound in this single-port method. But higher technology may be required to avoid compromising the brightness and resolution.

## Conclusion

As shown by our experience, a single-port approach is feasible and effective for rib resection. This simple method could expand the indications of single-port thoracoscopic surgery.

## Consent

Written informed consent was obtained from the patient for publication of this Case report and any accompanying images. A copy of the written consent is available for review by the Editor-in-Chief of this journal.

## Abbreviations

CT: Computed tomography.

## Competing interests

The authors declare that they have no competing interests.

## Authors’ contributions

CLH and BYW are the doctors for the primary care of the patients. CLH wrote the article. CYC and BYW performed the surgery and assisted for the postoperative care. CHL is the consultant of performing the study. All authors read and approved the final manuscript.
